# Osteopontin - The stirring multifunctional regulatory factor in multisystem aging

**DOI:** 10.3389/fendo.2022.1014853

**Published:** 2022-12-22

**Authors:** Yuxiang Du, Liwei Mao, Zhikun Wang, Kai Yan, Lingli Zhang, Jun Zou

**Affiliations:** School of Exercise and Health, Shanghai University of Sport, Shanghai, China

**Keywords:** osteopontin, multi-tissue regulation, cell senescence, osteoporosis, neurodegeneration

## Abstract

Osteopontin (OPN) is a multifunctional noncollagenous matrix phosphoprotein that is expressed both intracellularly and extracellularly in various tissues. As a growth regulatory protein and proinflammatory immunochemokine, OPN is involved in the pathological processes of many diseases. Recent studies have found that OPN is widely involved in the aging processes of multiple organs and tissues, such as T-cell senescence, atherosclerosis, skeletal muscle regeneration, osteoporosis, neurodegenerative changes, hematopoietic stem cell reconstruction, and retinal aging. However, the regulatory roles and mechanisms of OPN in the aging process of different tissues are not uniform, and OPN even has diverse roles in different developmental stages of the same tissue, generating uncertainty for the future study and utilization of OPN. In this review, we will summarize the regulatory role and molecular mechanism of OPN in different tissues and cells, such as the musculoskeletal system, central nervous system, cardiovascular system, liver, and eye, during senescence. We believe that a better understanding of the mechanism of OPN in the aging process will help us develop targeted and comprehensive therapeutic strategies to fight the spread of age-related diseases.

## 1 Introduction

With the extension of the average lifespan of a human, the quality of life of the elderly has not improved correspondingly because senile diseases have always plagued the elderly population. Aging is an extremely complex and heterogeneous biological process that involves almost every organ and tissue of the human body and is currently difficult to define clearly. Aging is characterized by a decrease in cell number and activity and a significant increase in the risk of disease (1). Neurodegenerative diseases caused by the aging of the nervous system have become one of the major causes of death (2). Magnetic resonance imaging (MRI) scans show that the brain volume decreases by 1% to 5% per year during aging, especially in areas responsible for memory and cognition (3). Sensory and motor nerve conduction velocities decrease substantially with age (4, 5). Muscle and bone loss may be devastating to exercise performance, and loss of exercise directly or indirectly accelerates the aging process. Sarcopenia, osteoporosis and osteoarthritis are typical diseases associated with musculoskeletal system aging. The senescence-associated secretory phenotype (SASP) disrupts the dynamic balance between osteoblast-mediated bone formation and osteoclast-mediated bone resorption (6). Cardiovascular diseases (coronary artery disease, hypertension, arrhythmia, stroke, atherosclerosis and cardiac insufficiency) are the most prominent diseases of aging, with ventricular wall hypertrophy and thickening, myocardial fibrosis, vascular sclerosis and thickening, and endothelial dysfunction are typical pathological manifestations (7). Lung structure and function change considerably with advancing age. The forced expiratory volume (FEV1) decreases by approximately 30 mL/year forced vital capacity (FVC) decreases by approximately 20 mL/year even in healthy people, and age-related chronic obstructive pulmonary disease (COPD) kills at least 3 million people a year (8, 9). The aging of the genitourinary system and digestive system also increases the strain on the body. Hormone levels are disrupted by atrophy and fibrosis of the ovaries and testes (10–12). Digestive dysfunction and intestinal flora disorder not only lead to nutrient absorption disorders but also affect many extralimentary systems (13–15). Although aging aging is difficult to reverse, prolonging life, avoiding the invasion of age-related diseases and pursuing healthy aging have always been some of the ultimate goals of scientific research.

Oldberg et al. (16) inferred the primary structure of a bone phosphoprotein (also called sialoprotein I) from cDNA cloned from rat osteosarcoma in 1986 and named it osteopontin (OPN). In 1989, the amino acid sequences encoded by the OPN cDNA secreted by mice, pigs and humans were successively isolated (17–19). Initially, OPN was proposed to exhibit a bone tissue-specific pattern and was expressed at high levels in the bone matrix. Subsequently, ectopic antigenic reactions of OPN were also detected in kidney and nerve tissues, suggesting that OPN has multiple origins (20, 21).

**Figure 1:**
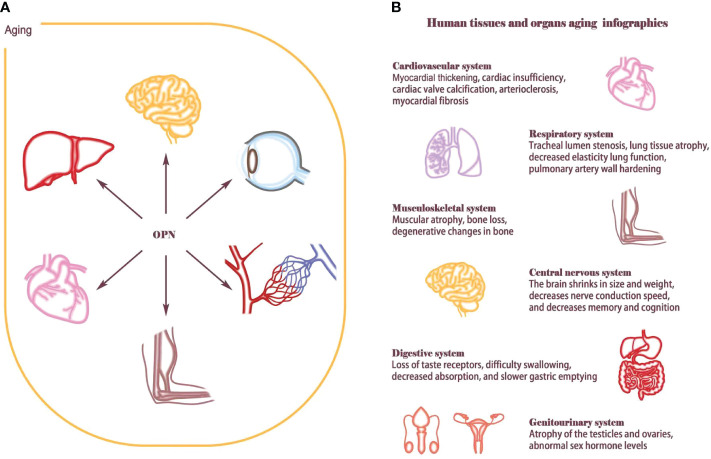
Aging affects human tissues and organs. **(A)** OPN is widely involved in the aging process of multiple organ tissues, such as T-cell senescence, atherosclerosis, skeletal muscle regeneration, osteoporosis, neurodegenerative changes, hematopoietic stem cell reconstruction and retinal aging. **(B)** Aging is an extremely complex and heterogeneous biological process that involves almost every organ and tissue of the human body. Aging causes cardiovascular diseases such as myocardial thickening, cardiac insufficiency, cardiac valve calcification, arteriosclerosis, and myocardial fibrosis; Lung structure and function change considerably with advancing age, such as tracheal lumen stenosis, lung tissue atrophy, decreased elasticity lung function, pulmonary artery wall hardening. Musculoskeletal system will appear muscle decreases, bone loss, bone degeneration. The central nervous system shrinks in size and weight, decreases nerve conduction speed, and decreases memory and cognition; The aging of the genitourinary system and digestive system also increases the load on the body. Hormone levels are disrupted by atrophy and fibrosis of the ovaries and testes, Digestive system dysfunction and intestinal flora imbalance eventually lead to nutrient absorption disorders.

OPN is involved in physiological and pathological processes and regulates the aging of various tissues and organs. However, the regulatory role and mechanism of OPN in the aging process of different tissues are not uniform. Here, we summarize the role and mechanism of OPN in mediating aging in different tissues and cells, providing ideas for further elucidating the secrets of aging (**Figure 1**).

## 2 Biogenesis and function of OPN

OPN is encoded by the secreted phosphoprotein 1 (SPP1) gene, a member of the small integrin-binding ligand N-linked glycoprotein (SIBLING) family along with four other genes, dentin matrix protein 1 (DMP1), dentin sialophosphoprotein (DSPP), integrin-binding sialoprotein (IBSP), and matrix extracellular phosphoglycoprotein (MEPE) (22). SIBLINGs are codirectional tandem genes located within the 37.5 kb region of human chromosome 4q21–25, and the SPP1 gene spans 7.8 kb in length with seven exons (23). The sixth exon is an exposed arginine-glycine-aspartate (RGD) domain that typically encodes more than 80% of the OPN protein.

OPN is a highly negatively charged secreted protein, and one-quarter of its residues are acidic. Its mode of interaction with calcium oxalate monohydrate and hydroxyapatite crystals involves the coordination of divalent cations with negatively charged residues on the crystal surface, and studies have shown that OPN is phosphorylated by the secretory kinase Fam20C (24). OPN is expressed in a variety of extracellular matrices (ECM) and regulates ECM remodeling (25). OPN is involved in a variety of pathophysiological processes, including bone metabolism, immune cell activation, cell migration, adhesion, and inhibition of apoptosis (26, 27). These regulatory effects of OPN are mainly derived from three receptor domains. The RGD domain binds to αvβ_1, 3, 5, 6, 8_, α_5, 8_β_1_, and α_IIβ_β_3_ integrins (26, 28). SVVYGLR and nonconserved ELVTDFPTDLPAT domains engage α_9_β_1_, α_4_β_7_ and α_4_β_1_ integrins (29, 30). The SVVYGLR sequence immediately follows the carboxyl terminus of RGD and is exposed at the carboxyl terminus of the N-terminal fragmented OPN after thrombin cleavage (31). In addition, full-length human OPN also contains a set of heparin-binding domains and calcium-binding domains that are recognized by splice variants of CD44, phosphorylation and O-glycosylation modification sites that determine the molecular weight, and two metalloproteinase (MMP) cleavage sites. The selective splicing of the human SPP1 pre-mRNA produces, five OPN isoforms (OPN-a, OPN-b, OPN-c, OPN-4, and OPN-5). These different isomers show specific expression in different cellular environments and have different biological functions. Existing studies have repoeted high OPN expression in osteoblasts, osteoclasts, vascular cells, smooth muscle cells, skeletal muscle cells, lymphocytes, endothelial cells, nerve cells and some carcinoma cells (32).

## 3 Role of OPN in musculoskeletal aging

Skeletal muscle aging is mainly manifested by an early decline in muscle strength and subsequent muscle atrophy due to impaired skeletal muscle protein synthesis. After age 40, skeletal muscle aging becomes apparent and affects activities of daily living (33). Cellular processes of protein synthesis and decomposition control changes in muscle mass. When the rate of protein degradation exceeds the rate of protein synthesis, protein and muscle fibers are impaired, resulting in skeletal muscle atrophy (34). Some studies have shown no abnormality in the balance between muscle anabolism and catabolism during healthy aging; however, the response of the body to some anabolic stimuli, such as nutritional supplementation and exercise, is significantly reduced, which is called anabolic resistance (35). In the process of pathological aging, the factors affecting skeletal muscle metabolism, including autophagy, muscle stem cell decline, noncoding RNA regulation, protein homeostasis disruption, skeletal muscle mitochondrial reduction, oxidative stress response, and insulin resistance, are more intricate, and these factors interact (36–42).

OPN plays an important regulatory role in a variety of processes, such as skeletal muscle cell proliferation, differentiation and regeneration. OPN is a key inflammatory cytokine involved in tissue remodeling (43). Various types of inflammatory cells express OPN, including T cells, neutrophils, and macrophages. OPN regulates the immune response by stimulating the expression of proinflammatory Th1-type cytokines and matrix-degrading enzymes (44). OPN expression is relatively low in normal muscles, but when muscle injury occurs, OPN expression increases approximately 120-fold above baseline within 12–24 h (43). Uaesoontrachoon et al. (45) found that OPN might support the rapid recovery of muscle function to normal in the early stage after injury. Limited acute OPN overexpression is beneficial to damaged muscle, but chronic OPN overexpression may lead to chronic damage, fibrosis and functional impairment of the damaged muscle (46). According to Vetrone et al. (47), the T lymphocyte subtype Vβ 8.1/8.2 isolated from a mouse muscular dystrophy model secretes OPN, suggesting that OPN is involved in inflammatory responses during muscle regeneration. OPN promotes muscular fibrosis in malnourished mice by regulating immune cell subsets and intramuscular TGF-β levels. A subsequent study found that OPN expression was increased in intramuscular inflammatory macrophages and the serum and myofiber niche of aged injured mice (48). Transwell assays of muscle stem cells and CD11b+ cells verified that an age-specific increase in OPN expression inhibited the muscle regeneration response. Injection of an OPN neutralizing antibody enhanced the myogenic response in elderly mice, while injection of the recombinant OPN protein inhibited muscle repair in young mice.

The process of bone growth is most active in childhood and adolescence, and bones are constantly being reshaped with increasing age. Disruption in the balance of processes of bone remodeling leads to disordered bone metabolism in the process of aging. OPN is an important component of the mineralized ECM of bones and teeth. It was first shown to be expressed at high levels in osteocytes, osteoblasts and osteoclasts. It serves as a signaling molecule for osteoblasts downstream of BMPs and stimulates the proliferation and calcification of osteoblasts (49). The OPN content is significantly higher in bone lines and periosteal plates during bone reconstruction. Researchers have suggested that OPN may play an important role in the adhesion process of OBs and the start and end of the bone metabolism cycle. In addition, OPN promotes osteoclast production and osteoclast activity through cellular signal transduction mediated by CD44 and αvβ3, thus participating in bone remodeling (50). Compared with old bones, higher levels of OC and OPN were detected in young bones (51). Prospective studies showed that the serum OPN level in menopausal women is significantly higher than that in women in the child-bearing period, and a negative correlation was observed between serum OPN levels and hip T-scores (52). Phosphorylation of bone matrix protein and OPN decreased by 20% and 30%, respectively, with age, leading to increased bone fragility (53). Another study showed that OPN^-/-^ young mouse bone properties at the nanoscale (elasticity and hardness) were significantly lower than those in wild-type mice, suggesting that OPN plays a role in promoting osteoblast differentiation during early bone formation. Interestingly, the mechanical properties of OPN^+/+^ mice decreased significantly with age, while the bone hardness and elasticity of OPN^-/-^ mice showed little change (54). Based on these results, OPN may play different roles in different stages of bone development. In mice with natural aging-induced osteoarthritis, OPN knockout leads to proteoglycan loss and increased MMP-13 release, resulting in decreased chondrocyte numbers and subchondral osteosclerosis (55).

**Figure 2:**
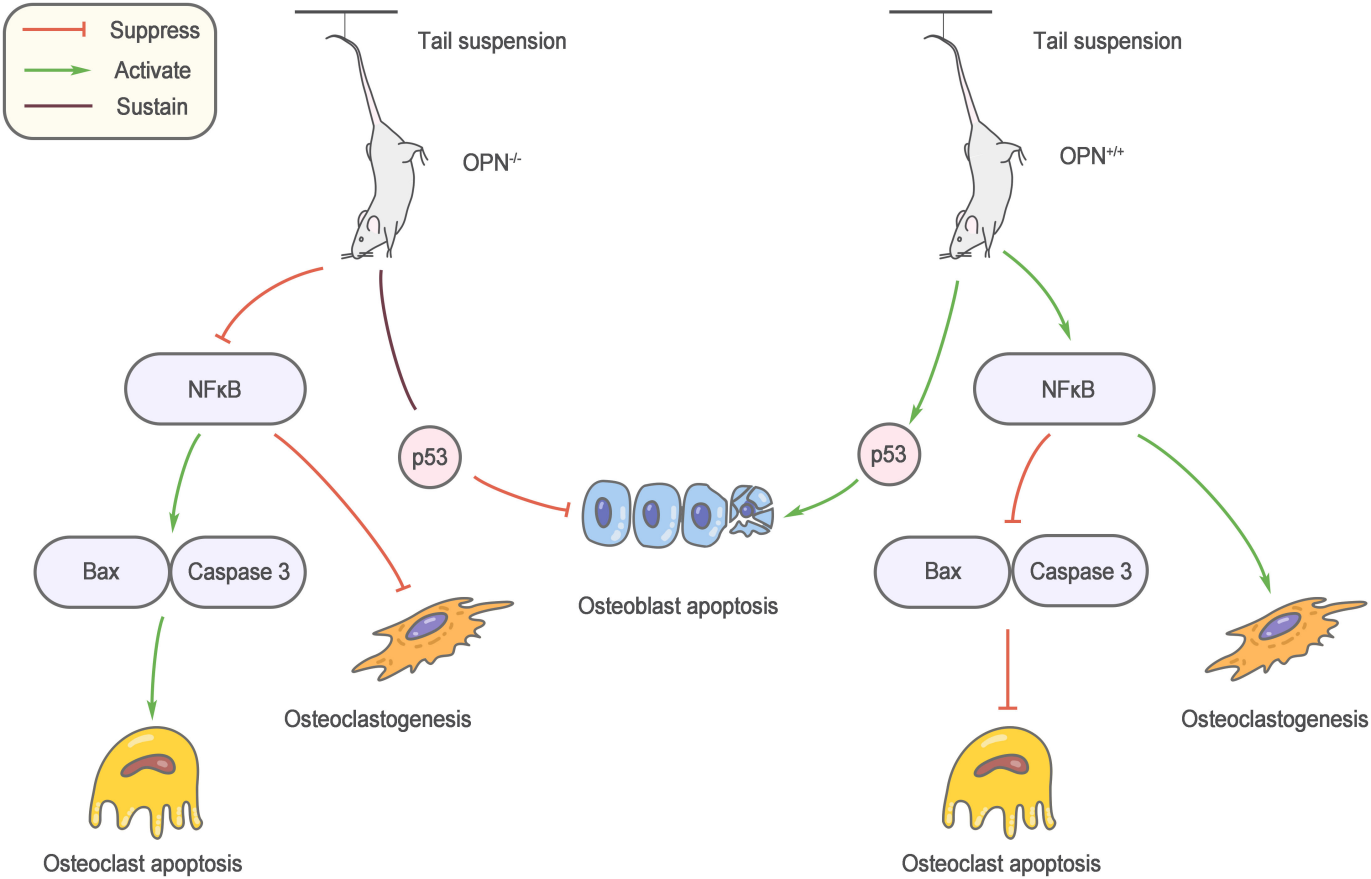
Role of OPN in musculoskeletal aging. OPN knockout affects multiple osteoblast and osteoclast regulatory factors during suspension, and nuclear factor kappa B (NF-κB)-mediated osteoclastogenesis and apoptosis inhibition are regulated by OPN.

Mechanical stimulation is an important factor regulating bone metabolism, and bone loss caused by unloading is an important cause of osteoporosis in elderly individuals. Ishijima et al. (56) found that the increase in bone resorption and reduction in bone formation induced by the mouse tail suspension model did not occur after OPN knockout. Specifically, the number of osteoclasts did not increase, and osteoblast-mediated bone formation was not affected. This result reveals a decisive role for OPN in bone loss due to unloading. OPN knockout affects multiple osteoblast and osteoclast regulatory factors during suspension, and nuclear factor kappaB (NF-κB)-mediated osteoclastogenesis and apoptosis inhibition are regulated by OPN, which may partly explain the anti-bone loss effect of OPN knockout (57) (**Figure 2**).

## 4 Role of OPN in central nervous system aging

With aging, the central nervous system gradually develops structural and functional deterioration, including gray matter and white matter damage, cortical atrophy, dysfunctional neurogenesis, a decreased brain volume, decreased nerve conduction velocity, and dysfunction of functional connectivity and cognition (58–60). Neurodegenerative diseases (NDDs) are incurable diseases related to aging that target different neuron groups in the central nervous system (CNS). Common senile NDDs include Alzheimer’s disease (AD), Parkinson’s disease (PD), Huntington’s disease (HD), and amyotrophic lateral sclerosis (ALS). Existing studies have shown that neuroinflammation, endoplasmic reticulum stress, oxidative stress, neuroimmune deficiency and mitochondrial dysfunction are involved in the physiological and pathological process of NDDs (61–65). Macrophage-, astrocyte- and microglia-mediated changes in neuroimmune function play important roles in NDDs, and impaired autophagy of neuronal cells results in the cumulative aggregation of amyloid plaques around presynaptic and perinuclear regions in the CNS (66–68). In addition, vascular aging, osteoporosis, and intestinal flora disorders are also involved in cross-organ regulation (60, 69–72).

Interestingly, existing studies support a strong correlation between acute and chronic NDDs and brain OPN levels in a time-dependent and age-dependent manner (73–75).

### 4.1 Relationship between OPN and PD

In several studies, plasma OPN levels in PD patients were closely associated with C-reactive protein levels, dyskinesia, and Hoehn-Yahr staging, based on examinations of plasma and cerebrospinal fluid for bone-derived factors (76, 77).

The inflammatory response may be the potential mechanism of OPN-induced PD. In an animal model of PD induced by 1-methyl-4-phenyl-1,2,3,6-tetrahydropyridine (MPTP), OPN knockout mice also showed less substantia nigra cell death and a reduced glial reaction (77, 78). Thus, OPN seems to play a role in facilitating PD progression. However, some studies have reached different conclusions. Iczkiewicz et al. (79) observed reduced OPN protein expression in surviving dopaminergic neurons of subjects with PD and was present in activated microglia but not in astrocytes. In addition, they also observed a decrease in OPN expression in the brains of two other patients with atypical PD disease associated with the loss of dopaminergic neurons (multiple system atrophy and progressive supranuclear palsy). Furthermore the RGD domain of OPN protects tyrosine hydroxylase (TH)-positive cells from MPP+- and LPS-induced toxic damage and protects against brain injury in the ventral region by increasing the levels of glial-derived neurotrophic factor (GDNF) and brain-derived neurotrophic factor (BDNF) and reducing the activation of glial cells (80, 81). Based on this information, OPN is closely related to dopaminergic neurons, and it exerts a protective effect on dopaminergic cells, which also indicates the potential role for OPN in neuroprotection, while the effect of OPN decreases with age, which may be a major factor inducing PD.

In conclusion, existing studies indicate that OPN may function as a double-edged sword that triggers neuronal toxicity or mediates neuroprotection in individuals with PD. More studies are needed to reveal the role of OPN in PD in the future.

### 4.2 Relationship between OPN and AD

OPN is expressed at high levels in the cerebrospinal fluid of patients with early AD and is associated with cognitive impairment (82). Autopsy results from AD patients and matched healthy subjects showed that AD patients had obvious amyloid-beta protein (Aβ) deposition in the hippocampus, and OPN expression was significantly increased, which was positively correlated with age and the Aβ level (83).

In a recent cross-sectional study, researchers reported a significant association between higher OPN levels in the blood and AD and brain atrophy (84). In addition, in several animal models of AD, OPN mRNA and protein levels were also confirmed to be significantly upregulated (78, 85). As an immunomodulatory cytokine, the increase in OPN levels may be the body’s defense response to neurodegeneration with the role of eliminating pathogenic Aβ. At present, research has also shown that OPN is positively correlated with inflammatory cytokines. Rentsendor et al. (86) found that OPN increased the recruitment of monocyte-macrophages to the brain of ADtg mice and promoted the polarization of macrophages to an anti-inflammatory and highly phagocytic phenotype, thus regulating the inflammatory response of the nervous system during AD and promoting the clearance of Aβ.

OPN was shown to be widely expressed by infiltrating macrophages and microglia after demyelinating injury. The cumulative expression of OPN in aged mice after injury was lower than that in young mice, indicating that OPN played a certain but not decisive role in myelin regeneration (87). Kainic acid is a glutamic acid analog, and its excitatory toxicity induces hippocampal damage, causing the proliferation and activation of astrocytes and microglia. Sanae et al. (88) administered a kainic acid injection to induce hippocampal injury in mice and found that the expression levels of OPN and its receptor, CD44, were significantly upregulated in senescence-resistant mice, while a significant difference was not observed in senescence-accelerated mice. The upregulation of OPN is accompanied by the upregulation of CD44. OPN interacts with CD44 to form the OPN-CD44 complex, which exerts antiapoptotic activity. Due to the important role of the OPN-CD44 complex in neuroprotection and reconstruction, the increased OPN expression may represent a response to nerve injury (89). OPN is generally considered a proinflammatory cytokine, but more recent studies have shown that OPN is a promising neuroprotective agent that may help fight AD-related pathological processes and promote tissue repair in the brain.

### 4.3 Relationship between OPN and ALS

Amyotrophic lateral sclerosis (ALS) is a motor neuron disease characterized by the gradual death and loss of motor neurons in the central nervous system, resulting in patients gradually losing the ability to control autonomic movement. Patients often eventually die of respiratory or swallowing failure. Although few related studies have been conducted, preliminary studies have also reported the neuroprotective effect of OPN on ALS. Studies have confirmed that astrocytes dysfunction plays a vital role in the progression of the disease. Izrael et al. (90) induced human embryonic stem cells to differentiate into astrocytes and transplanted them into hSOD1^G93A^ transgenic mice and rats through an intramyelin injection. Animals in the treatment group exhibited a significantly delayed onset of ALS and improved motor performance compared to those in the control group. An analysis of the secretion group showed that the transplanted astrocytes secreted several metalloprotease inhibitors, including OPN, and several neuroprotective factors. Therefore OPN may become an effective therapy for ALS. They authors also suggested that the mechanism of action may be that OPN activates astrocytes and microglia through a noncell-autonomous action, that promotes the survival and regeneration of neurons, and then mediates neuroprotection. Based on these findings, OPN may become a potential target for the prevention and treatment of ALS.

In conclusion, we have found that OPN exhibits significant changes in the physiological and pathological processes of several neurodegenerative diseases and may be considered a potential target for the treatment of related diseases in the future, given its role in neuroinflammation.

## 5 Role of OPN in cardiovascular system aging

Aging, the greatest risk factor for cardiovascular disease, largely dominates the accumulation of changes in the structure and function of the cardiovascular system. Common aging-related cardiovascular diseases include atherosclerosis, hypertension, cardiovascular neurosis, myocardial infarction, cardiomyopathy and stroke, and related cardiovascular histopathological changes include cardiac hypertrophy, valvular calcification, myocardial fibrosis, arteriosclerosis, and impaired endothelial function.

### 5.1 OPN accelerates myocardial fibrosis related to aging

**Figure 3:**
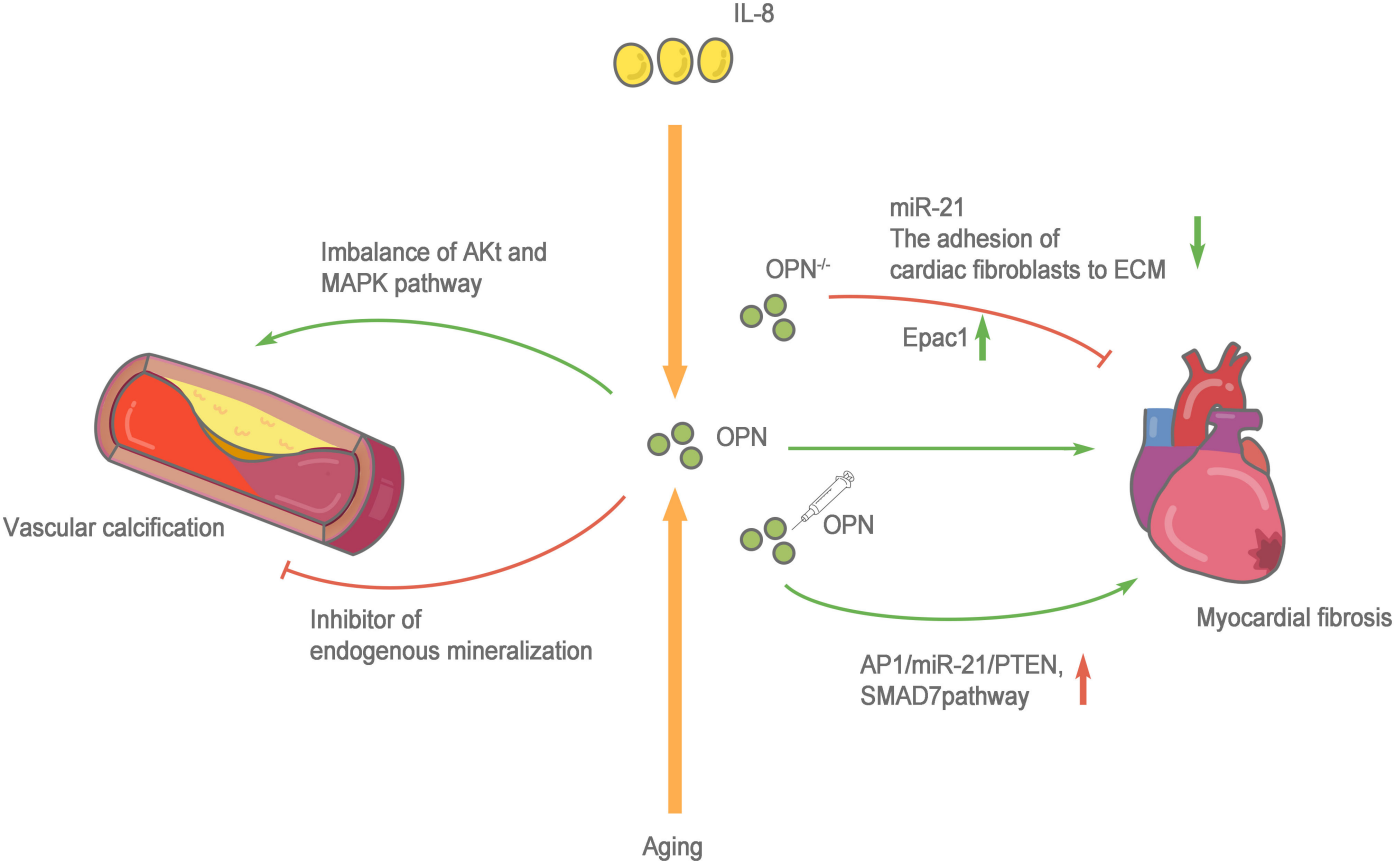
Role of OPN in cardiovascular system aging. The cytokine interleukin (IL)-18 and aging are involved in inducing OPN expression and promoting myocardial fibrosis and cardiac dysfunction. when OPN-/-, the adhesion of cardiac fibroblasts to extracellular matrices (ECM) is greatly reduced, the expression level of miR-21 decreases and β1AR and β_2_AR are activated to enhance the production of cAMP, increasing the protein level of Epac1 to combat the fibrosis of cardiac fibroblasts. Whereas an injection of the recombinant OPN protein reactivates the AP1/miR-21/PTEN and SMAD7 pathways and enhances fibrosis. OPN has two sides to vascular calcification, on the one hand, OPN activated the MAPK signaling pathway, and the imbalance of Akt and MAPK pathways leads to the switching of vascular smooth muscle cells (VSMCs) to pathological progression. on the other hand, OPN is an effective physiological inhibitor of endogenous mineralization, which can prevent ectopic calcium deposition, and is an effective inhibitor of vascular calcification.

Cardiac fibrosis is defined as a state of excessive extracellular deposition of collagen and ECM (91). During aging, progressive myocardial cell hypertrophy, inflammation, and the progressive development of cardiac fibrosis are important markers of cardiac aging (92). Myocardial fibrosis refers to the repair of the heart in response to injury, which is conducive to maintaining the structural integrity of the heart. However, due to the nonrenewable nature of myocardial cells, the damaged myocardial cells are replaced by ECM components, such as collagen. Collagen does not serve to replace the function of normal cardiomyocytes, and excessive deposition of collagen leads to cardiac dysfunction. In addition to the cardiomyocytes responsible for contraction and relaxation, the healthy heart is also composed of fibroblasts, endothelial cells, macrophages, and other cell types. Very few fibroblasts are present in a healthy heart. However, when the heart is damaged, fibroblasts differentiate into myofibroblasts, which secrete large amounts of collagen and other ECM proteins (93). Therefore, the functional state of fibroblasts in the heart plays an important role in the occurrence and progression of myocardial fibrosis. Normally, the heart only expresses low levels of OPN. However, several studies have confirmed that OPN expression increases significantly under a variety of pathological conditions (such as myocardial infarction and heart failure), and the infiltrated macrophages in the injury area are the main source of OPN (94). OPN is directly or indirectly involved in cardiac fibrosis through various mechanisms. OPN-regulated angiotensin-induced cardiac fibrosis and the adhesion of cardiac fibroblasts to ECM are substantially reduced after OPN knockout (95, 96). Endocardial biopsy in patients with dilated cardiomyopathy showed higher OPN expression than that in healthy people and was positively correlated with myocardial collagen accumulation and negatively correlated with the left ventricular ejection fraction (97). The cytokine interleukin (IL)-18 is involved in inducing OPN expression and promoting myocardial fibrosis and cardiac dysfunction (98, 99). Lorenzen et al. (100) confirmed that OPN was involved in the pathological process of myocardial fibrosis, the regulation of which depends on a noncoding RNA. OPN silencing reduces the expression level of miR-21 and cardiac fibrosis. An injection of the recombinant OPN protein reactivates the AP_1_/miR-21/PTEN and SMAD7 pathways and enhances fibrosis. OPN blockade increases cAMP generation by activating the β_1_ adrenergic receptor (AR) and β_2_AR on H9c2 cardiomyocytes to increase the Epac1 protein level and inhibit the fibrosis of cardiac fibroblasts. Furthermore, OPN inhibits β_2_AR cAMP signal transduction through a direct interaction with the GαS/olf protein subunit in H9c2 cardiomyocytes (101). In addition, the expression of OPN in fibroblasts is upregulated during granulocyte-myeloid suppressor cell (G-MDSC) derived S100A8/A9-induced cardiomyocyte inflammatory phenotypes (102). Sawaki et al. (103) found that visceral adipose tissue, which protects fibroblasts from senescence and accelerates cardiac senescence, is an important source of OPN. After removing visceral adipose tissue and OPN, mouse cardiac fibroblasts show substantially increased senescence and apoptosis. The CD153+PD-1+CD44hiCD4+ T cells produced by visceral fat secrete large amounts of OPN and PD-1 resistance, leading to inflammation and promoting aging (104). Lin et al. (105) observed significantly increased OPN expression in the peripheral circulation of patients with atrial fibrillation. In cell cultures *in vitro*, OPN promoted atrial fibrosis by activating the Akt/GSK-3β/β-catenin pathway and inhibiting autophagy. These studies confirmed that OPN, an indispensable comonent in another pathway, directly or indirectly protects cardiac fibroblasts and accelerates cardiac fibrotic aging, making it an important target for treatment and prevention (**Figure 3**).

### 5.2 OPN accelerates vascular calcification

Vascular aging refers to degenerative changes in the structure and function of vessels with age. Metabolic changes and calcification of blood vessels are the main characteristics of aging. Histologically, the changes in vascular endothelial and middle layer structure and function are mainly manifested as increases in connective tissue, lipid and calcium content in the lower layer of vascular endothelial cells, thickening of the vascular smooth muscle layer, a decrease in the elastin content, and the occurrence of vascular calcification (106, 107). Currently, known mechanisms of vascular aging mainly include oxidative stress, mitochondrial dysfunction, vascular inflammation, endothelial dysfunction, altered protein homeostasis, cellular phenotypic switching, apoptosis, and genomic instability (108). Vascular calcification regulates calcium deposition and thus interacts with bone matrix deposition and bone metabolism. OPN is an important mediator of bone matrix deposition and has recently been reported to be involved in the pathological process of vascular aging.

OPN is a regulatory protein involved in pathological calcification, and OPN expression is closely related to the process of vascular calcification. Mohler et al. (109) found that OPN was abundantly expressed in calcified human aortic valves and was colocated with calcified deposits in the valves. Similar to Aβ in the nervous system, macrophages expressing the OPN mRNA surround atherosclerotic plaques, and the level of OPN mRNA expression increases with the progression of atherosclerosis (110). Patients with chronic venous insufficiency (CVI) have elevated levels of osteogenic markers of the venous wall, including RUNX2, OCN and OPN, along with amorphous calcification, ossification, and fibrin deposition (111). OPN levels in circulating blood were also significantly higher in obese patients with vascular, vascular cognitive impairment and type 2 diabes, which may be closely related to vascular calcification (84, 112). In addition, the plasma OPN level in patients with coronary artery calcification also increased significantly (113). Thus, OPN exhibits good binding to calcium ions, and OPN binds many calcium ions with high affinity (24). Many studies have confirmed that OPN is an effective physiological inhibitor of endogenous mineralization, that prevents ectopic calcium deposition, and vascular calcification. *In vitro* studies have shown that OPN^-/-^ vascular smooth muscle cells (VSMCs) contain higher concentrations of inorganic phosphate (a vascular calcification inducer), and their calcification level is significantly higher than that of wild-type VSMCs (114). In addition, *in vivo* experiments have confirmed the protective effect of OPN on vascular calcification using various approaches (115–117). However, some studies have found that OPN is involved in the pathological process of vascular aging.

Zhang et al. (118) found that OPN significantly activated the MAPK signaling pathway during the transformation of VSMCs induced by aging and hypertension. The imbalance of Akt and MAPK pathways leads to the switching of VSMCs to proliferative and differentiated phenotypes and promotes pathological progression. Shao et al. (119) revealed the double-sided effect of OPN. OPN plays a stage-specific role in atherosclerosis in LDLR^-/-^ mice: OPN promotes calcification through the proinflammatory metabolite SVVYGLR at the early stage of diabetes and vascular disease, but OPN limits vascular cartilage-like metaplasia, endochondral mineralization and collagen accumulation at a later stage of disease progression to inhibit vascular calcification-induced damage. A complete lack of OPN leads to the aggravation of atherosclerotic disease. However, many studies have suggested that OPN has a dual role in the process of venous disease, and one of the reasons for this duality is the change in OPN phosphorylation with age (120). Notably, the phosphorylation of OPN is an important factor modulating its physiological function of regulating the calcification process (121). Furthermore, only phosphorylated OPN exerts a positive effect on inhibiting biomineralization (122, 123).

However, based on the existing research results, the molecular pathway mechanism of OPN in vascular calcification is not clear and requires further exploration (**Figure 3**).

### 5.3 OPN maintains hematopoietic stem cell reconstitution

The increase in the number of aged hematopoietic stem cells (HSCs) is due to a higher rate of self-renewal through cell division in conjunction with a reduced inherent ability to rebuild. During senescence, OPN deficiency is associated with an increase in the number of peripheral blood lymphocytes and a decrease in the erythrocyte numbers. Mice transplanted with OPN^-/-^ bone marrow stem cells die prematurely due to impaired red blood cell synthesis and thrombocytopenia (124). Guidi et al. (125) detected reduced levels of OPN in bone marrow in elderly mice, which led to a senescent phenotype in hematopoietic stem cells. OPN^-/-^ results in a severe decrease in transplantation and an increase in the long-term frequency of young HSCs. In contrast, exposure of elderly HSCs to thrombin‐cleaved OPN increased transplantation, reduced the HSC frequency, increased stem cell polarity, and reconstructed the balance between lymphocytes and bone marrow cells. In addition, the age-related OPN deficiency promotes the migration of HSCs to the liver, increases hepatic iron deposition, and increases inflammation and oxidative stress, which are important factors causing liver damage and decreased functional (126). OPN has been verified to be an important factor maintaining the structure and function of HSCs during both fetal development and aging.

## 6 OPN is involved in regulating liver aging and regeneration

Aging of the liver continually alters its morphological and physiological functions; it reduces liver volume, causes steatosis and fibrosis and reduces hepatic blood flow. During liver aging, hepatocytes are exposed to a series of adverse microenvironmental factors, including increased inflammation, endoplasmic reticulum oxidative stress (ER stress), and hepatocyte toxicity (127–130). Liver regeneration is an important biological activity that repairs liver function after injury. However, with the effects of aging, the large amounts of energy and cell material required for liver regeneration are insufficient, and liver regeneration is seriously damaged, leading to cell necrosis and apoptosis (131, 132).

Liver fibrosis is the representative pathological change of liver aging. Similar to the role of OPN in myocardial fibrosis, OPN also exerts a positive regulatory effect on liver fibrosis. Urtasun et al. (133) reported that OPN promoted hepatic fibrosis through hepatic stellate cell activation and ECM deposition. In addition, OPN delays the dissolution of fibrotic liver tissue by maintaining type I collagen fiber deposition (134). Chronic inflammation and oxidative stress are the main characteristics of the microenvironment of tissues and organs in the aging process, and are closely related to the aging and necrosis of liver cells.

OPN is an important promoter of liver regeneration. OPN promotes cell proliferation and reduces apoptosis during liver regeneration, whereas silencing OPN inhibits the liver regeneration rate. In a mouse model of partial hepatectomy, OPN knockout significantly delayed liver regeneration. A gene chip analysis showed that OPN was expressed at high levels in hepatocytes, pit cells, oval cells and bile duct epithelial cells during liver regeneration and participated in the proliferation, differentiation and migration of these cells (135). According to recent studies, OPN levels in the liver tissue of aged mice are decreased. Therefore, decreased OPN levels may be an important factor inducing an age-related decrease in liver regeneration. Autophagy is a vital cellular activity for liver regeneration that provides glucose, amino acids and free fatty acids to liver cells to sustain basic metabolic activities and promote the physiological restoration of the aging liver (136, 137). The autophagy activity of the aged liver is inferior to that of the young liver. An increasing number of studies have confirmed that the regenerative function of the aged liver is closely related to deficient autophagy (136, 138–141). Therefore, OPN has a complex dual role in the process of liver aging; it not only induces the development of age-related liver fibrosis but also delays the aging and apoptosis of hepatocytes and promotes the regeneration of hepatocytes through mechanisms such as autophagy (142). Thus, the relationship between OPN and liver aging remains a complex subject that deserves further investigation.

## 7 OPN is involved in aging-related eye diseases

Retinal neurodegeneration associated with aging is a common mechanism of senile eye diseases. In the DBA2/J (D2J) mouse model, a model of hereditary optic neuropathy, retinal OPN levels were significantly increased, and the content of OPN in plasma also increased significantly, especially in older mice (143). In individual mice with severe optic neuropathy, the cumulative OPN concentration increased dramatically. Subsequent studies revealed a significantly reduced number of OPN^+^ retinal ganglion cells (RGCs) in D2J mice, and OPN supplementation inhibited cellular degeneration in the ganglion cell layer and increased the metabolic activity of neuronal precursor cells ex vivo. The researchers proposed that this paradoxical result may be explained by the increased expression of OPN in the early stage of the disease that exerts a protective effect but that OPN accumulates as aging progresses and the disease progresses without intervention by counteractings the protective effect of OPN and even causing damage. Ruzafa et al. (144) found that in OPN^-/-^ mice, the density of RGCs and the surface area of astrocytes were significantly reduced, and a strong age-dependent effect was observed. In addition, the density of RGCs and astrocytes in the retinas of 3-month-old OPN^-/-^ mice was comparable to that of 20-month-old wild-type mice, suggesting that OPN deficiency may contribute to premature retinal aging in mice. However, OPN was identified as a basal sediment component in the retina of another eye disease, age-related macular degeneration (AMD), with a distinctive spot-like staining pattern and colocalization with abnormal calcium deposits (145).

Based on current information, OPN plays a dual role in aging-related eye diseases. On the one hand, in the early stage of the disease, increased OPN expression plays a protective role to counter adverse pathological changes; on the other hand, the excessive accumulation of OPN aggravates calcification and deposition in tissues, providing an important link to the pathological degeneration of aging-related eye diseases.

## 8 Role of OPN and immune aging

“Immune aging” refers to the changes in innate and adaptive immune functions that occur in the elderly as the function of the immune system gradually declines with age (146). Immune aging is mainly reflected in impaired T-cell function, which leads to an increase in the body’s susceptibility to infectious diseases and cancers (147). Studies have confirmed that the aging of CD4+ T-cell immunity is an important reason for the changes that occur in immune aging (148). According to current research, the senescence-associated secretory phenotype (SASP) is one of the important mechanisms of cellular aging and that the secretion of a large number of proinflammatory factors is considered a key feature of the SASP. Studies have documented a significant increase in OPN expression in aging CD4+ T cells (149). Xiong et al. (150, 151) used the animal model of D-galactose-induced aging, and the OPN mRNA level in CD4+ T cells also increased significantly. In addition, they cocultured CD4+ T cells with human placental mesenchymal stem cells (hPMSCs) or treated CD4+ T cells with exosomes derived from hPMSCs, and both of these experiments showed an improvement of the aging phenotype of CD4+ T cells and downregulation of the OPN mRNA level. In summary, OPN is an important type I cytokine, and the level of its secretion increases with age. In addition, OPN is also a component of the SASP, which mediates the aging and functional decline of cells and the immune system. Therefore, the level of OPN may be used as a biomarker of immune aging and as a potential therapeutic target.

## 9 Conclusion

Although OPN has been identified to participate in the aging process of many tissues, the interaction between tissues and organs has not been studied. Aging is a complex process involving all body systems. Further studies are needed to determine whether and how OPN participates in cross-organ regulation.

At present, most studies have adopted OPN systemic knockout, and the study of tissue-specific conditional knockout animals may provide a wider field of research. In addition, numerous research results have shown the dual roles of OPN in the aging process. For example, in the nervous system, OPN not only causes neurotoxicity but also acts as a neuroprotective agent for PD. In the process of liver aging, OPN not only induces the occurrence and development of age-related liver fibrosis but also delays the aging and apoptosis of hepatocytes and promotes their regeneration by restoring the autophagy activity of aging liver cells. In addition, OPN also plays a dual role in the process of eye aging. On the one hand, in the early stage of the disease, OPN expression increases to mediate its protective effect on adverse pathological changes. On the other hand, excessive accumulation of OPN aggravates calcification and calcium deposition in tissues, which is an important link in pathological degeneration. The role of OPN in the aging process has not been fully clarified. More importantly, the critical point of this phenomenon is not completely clear. Therefore, further in-depth studies investigating whether OPN mediates and participates in the effects of antiaging factors such as sports, nutrition and healthy lifestyle on the aging process are worthwhile and will hopefully provide new ideas and treatment schemes for many clinical diseases in the future.

## Author contributions

JZ gave the brief introduction to this article. YD, LM, ZW, and KY were responsible for manuscript writing. LZ revised the manuscript. All authors approved the final version of this manuscript.

## References

[1] Morris A. Ageing: Are the secrets of healthy ageing within 'young blood'? Nat Rev Endocrinol (2017) 13(7):376. doi: 10.1038/nrendo.2017.60 28474688

[2] Erkkinen MG Kim MO Geschwind MD. Clinical neurology and epidemiology of the major neurodegenerative diseases. Cold Spring Harb Perspect Biol (2018) 10(4):a03318. doi: 10.1101/cshperspect.a033118 PMC5880171 28716886

[3] Scheiblich H Trombly M Ramirez A Heneka MT. Neuroimmune connections in aging and neurodegenerative diseases. Trends Immunol (2020) 41(4):300–12. doi: 10.1016/j.it.2020.02.002 32147113

[4] Wang TY Chen SC Peng CW Kang CW Chen YL Chen CL. Relevance of nerve conduction velocity in the assessment of balance performance in older adults with diabetes mellitus. Disabil Rehabil (2017) 39(5):419–27. doi: 10.3109/09638288.2016.1146352 26937553

[5] Walsh ME Sloane LB Fischer KE Austad SN Richardson A Van Remmen H. Use of nerve conduction velocity to assess peripheral nerve health in aging mice. J Gerontol A Biol Sci Med Sci (2015) 70(11):1312–9. doi: 10.1093/gerona/glu208 PMC4612382 25477428

[6] Baar MP Perdiguero E Muñoz-Cánoves P de Keizer PL. Musculoskeletal senescence: a moving target ready to be eliminated. Curr Opin Pharmacol (2018) 40:147–55. doi: 10.1016/j.coph.2018.05.007 29883814

[7] Abdellatif M Sedej S Carmona-Gutierrez D Madeo F Kroemer G. Autophagy in cardiovascular aging. Circ Res (2018) 123(7):803–24. doi: 10.1161/circresaha.118.312208 30355077

[8] Cho SJ Stout-Delgado HW. Aging and lung disease. Annu Rev Physiol (2020) 82:433–59. doi: 10.1146/annurev-physiol-021119-034610 PMC7998901 31730381

[9] Rabe KF Watz H. Chronic obstructive pulmonary disease. Lancet (2017) 389(10082):1931–40. doi: 10.1016/s0140-6736(17)31222-9 28513453

[10] Gunes S Hekim GN Arslan MA Asci R. Effects of aging on the male reproductive system. J Assist Reprod Genet (2016) 33(4):441–54. doi: 10.1007/s10815-016-0663-y PMC4818633 26867640

[11] Hommos MS Glassock RJ Rule AD. Structural and functional changes in human kidneys with healthy aging. J Am Soc Nephrol (2017) 28(10):2838–44. doi: 10.1681/asn.2017040421 PMC5619977 28790143

[12] Zhang J Chen Q Du D Wu T Wen J Wu M. Can ovarian aging be delayed by pharmacological strategies? Aging (2019) 11(2):817–32. doi: 10.18632/aging.101784 PMC6366956 30674710

[13] Quigley EMM. Microbiota-Brain-Gut axis and neurodegenerative diseases. Curr Neurol Neurosci Rep (2017) 17(12):94. doi: 10.1007/s11910-017-0802-6 29039142

[14] Jin M Qian Z Yin J Xu W Zhou X. The role of intestinal microbiota in cardiovascular disease. J Cell Mol Med (2019) 23(4):2343–50. doi: 10.1111/jcmm.14195 PMC6433673 30712327

[15] Tang WHW Li DY Hazen SL. Dietary metabolism, the gut microbiome, and heart failure. Nat Rev Cardiol (2019) 16(3):137–54. doi: 10.1038/s41569-018-0108-7 PMC6377322 30410105

[16] Oldberg A Franzén A Heinegård D. Cloning and sequence analysis of rat bone sialoprotein (osteopontin) cDNA reveals an arg-Gly-Asp cell-binding sequence. Proc Natl Acad Sci U.S.A. (1986) 83(23):8819–23. doi: 10.1073/pnas.83.23.8819 PMC387024 3024151

[17] Miyazaki Y Setoguchi M Yoshida S Higuchi Y Akizuki S Yamamoto S. Nucleotide sequence of cDNA for mouse osteopontin-like protein. Nucleic Acids Res (1989) 17(8):3298. doi: 10.1093/nar/17.8.3298 2726465 PMC317738

[18] Kiefer MC Bauer DM Barr PJ. The cDNA and derived amino acid sequence for human osteopontin. Nucleic Acids Res (1989) 17(8):3306. doi: 10.1093/nar/17.8.3306 2726470 PMC317745

[19] Wrana JL Zhang Q Sodek J. Full length cDNA sequence of porcine secreted phosphoprotein-I (SPP-I, osteopontin). Nucleic Acids Res (1989) 17(23):10119. doi: 10.1093/nar/17.23.10119 2602123 PMC335255

[20] Mark MP Prince CW Gay S Austin RL Butler WT. 44-kDal bone phosphoprotein (osteopontin) antigenicity at ectopic sites in newborn rats: Kidney and nervous tissues. Cell Tissue Res (1988) 251(1):23–30. doi: 10.1007/bf00215443 3277715

[21] Yoon K Buenaga R Rodan GA. Tissue specificity and developmental expression of rat osteopontin. Biochem Biophys Res Commun (1987) 148(3):1129–36. doi: 10.1016/s0006-291x(87)80250-4 3500718

[22] Bellahcène A Castronovo V Ogbureke KU Fisher LW Fedarko NS. Small integrin-binding ligand n-linked glycoproteins (SIBLINGs): multifunctional proteins in cancer. Nat Rev Cancer (2008) 8(3):212–26. doi: 10.1038/nrc2345 PMC2484121 18292776

[23] Bouleftour W Juignet L Verdière L Machuca-Gayet I Thomas M Laroche N. Deletion of OPN in BSP knockout mice does not correct bone hypomineralization but results in high bone turnover. Bone (2019) 120:411–22. doi: 10.1016/j.bone.2018.12.001 30529011

[24] Klaning E Christensen B Sorensen ES Vorup-Jensen T Jensen JK. Osteopontin binds multiple calcium ions with high affinity and independently of phosphorylation status. Bone (2014) 66:90–5. doi: 10.1016/j.bone.2014.05.020 24928493

[25] Kazanecki CC Uzwiak DJ Denhardt DT. Control of osteopontin signaling and function by post-translational phosphorylation and protein folding. J Cell Biochem (2007) 102(4):912–24. doi: 10.1002/jcb.21558 17910028

[26] Yokosaki Y Tanaka K Higashikawa F Yamashita K Eboshida A. Distinct structural requirements for binding of the integrins alphavbeta6, alphavbeta3, alphavbeta5, alpha5beta1 and alpha9beta1 to osteopontin. Matrix Biol (2005) 24(6):418–27. doi: 10.1016/j.matbio.2005.05.005 16005200

[27] Briones-Orta MA Avendaño-Vázquez SE Aparicio-Bautista DI Coombes JD Weber GF Syn WK. Osteopontin splice variants and polymorphisms in cancer progression and prognosis. Biochim Biophys Acta Rev Cancer (2017) 1868(1):93–108.a. doi: 10.1016/j.bbcan.2017.02.005 28254527

[28] Barry ST Ludbrook SB Murrison E Horgan CM. A regulated interaction between alpha5beta1 integrin and osteopontin. Biochem Biophys Res Commun (2000) 267(3):764–9. doi: 10.1006/bbrc.1999.2032 10673366

[29] Bayless KJ Davis GE. Identification of dual alpha 4beta1 integrin binding sites within a 38 amino acid domain in the n-terminal thrombin fragment of human osteopontin. J Biol Chem (2001) 276(16):13483–9. doi: 10.1074/jbc.M011392200 11278897

[30] Ito K Kon S Nakayama Y Kurotaki D Saito Y Kanayama M. The differential amino acid requirement within osteopontin in alpha4 and alpha9 integrin-mediated cell binding and migration. Matrix Biol (2009) 28(1):11–9. doi: 10.1016/j.matbio.2008.10.002 19000758

[31] Yokosaki Y Matsuura N Sasaki T Murakami I Schneider H Higashiyama S. The integrin alpha(9)beta(1) binds to a novel recognition sequence (SVVYGLR) in the thrombin-cleaved amino-terminal fragment of osteopontin. J Biol Chem (1999) 274(51):36328–34. doi: 10.1074/jbc.274.51.36328 10593924

[32] Han X Wang W He J Jiang L Li X. Osteopontin as a biomarker for osteosarcoma therapy and prognosis. Oncol Lett (2019) 17(3):2592–8. doi: 10.3892/ol.2019.9905 PMC6365895 30854034

[33] Moro T Ebert SM Adams CM Rasmussen BB. Amino acid sensing in skeletal muscle. Trends Endocrinol Metab (2016) 27(11):796–806. doi: 10.1016/j.tem.2016.06.010 27444066 PMC5075248

[34] Cohen S Nathan JA Goldberg AL. Muscle wasting in disease: molecular mechanisms and promising therapies. Nat Rev Drug Discovery (2015) 14(1):58–74. doi: 10.1038/nrd4467 25549588

[35] Rennie MJ. Anabolic resistance: the effects of aging, sexual dimorphism, and immobilization on human muscle protein turnover. Appl Physiol Nutr Metab (2009) 34(3):377–81. doi: 10.1139/h09-012 19448702

[36] Park SS Seo YK Kwon KS. Sarcopenia targeting with autophagy mechanism by exercise. BMB Rep (2019) 52(1):64–9. doi: 10.5483/BMBRep.2019.52.1.292 PMC6386226 30526769

[37] Henze H Jung MJ Ahrens HE Steiner S von Maltzahn J. Skeletal muscle aging - stem cells in the spotlight. Mech Ageing Dev (2020) 189:111283. doi: 10.1016/j.mad.2020.111283 32544406

[38] Jung HJ Lee KP Kwon KS Suh Y. MicroRNAs in skeletal muscle aging: Current issues and perspectives. J Gerontol A Biol Sci Med Sci (2019) 74(7):1008–14. doi: 10.1093/gerona/gly207 PMC6580686 30215687

[39] Fernando R Drescher C Nowotny K Grune T Castro JP. Impaired proteostasis during skeletal muscle aging. Free Radic Biol Med (2019) 132:58–66. doi: 10.1016/j.freeradbiomed.2018.08.037 30194981

[40] Johnson ML Robinson MM Nair KS. Skeletal muscle aging and the mitochondrion. Trends Endocrinol Metab (2013) 24(5):247–56. doi: 10.1016/j.tem.2012.12.003 PMC3641176 23375520

[41] Shou J Chen PJ Xiao WH. Mechanism of increased risk of insulin resistance in aging skeletal muscle. Diabetol Metab Syndr (2020) 12:14. doi: 10.1186/s13098-020-0523-x 32082422 PMC7014712

[42] McDonagh B Sakellariou GK Jackson MJ. Application of redox proteomics to skeletal muscle aging and exercise. Biochem Soc Trans (2014) 42(4):965–70. doi: 10.1042/bst20140085 25109987

[43] Hoffman EP Gordish-Dressman H McLane VD Devaney JM Thompson PD Visich P. Alterations in osteopontin modify muscle size in females in both humans and mice. Med Sci Sports Exerc (2013) 45(6):1060–8. doi: 10.1249/MSS.0b013e31828093c1 PMC3631433 23274598

[44] Zanotti S Gibertini S Di Blasi C Cappelletti C Bernasconi P Mantegazza R. Osteopontin is highly expressed in severely dystrophic muscle and seems to play a role in muscle regeneration and fibrosis. Histopathology (2011) 59(6):1215–28. doi: 10.1111/j.1365-2559.2011.04051.x 22175901

[45] Uaesoontrachoon K Wasgewatte Wijesinghe DK Mackie EJ Pagel CN. Osteopontin deficiency delays inflammatory infiltration and the onset of muscle regeneration in a mouse model of muscle injury. Dis Model Mech (2013) 6(1):197–205. doi: 10.1242/dmm.009993 22917925 PMC3529351

[46] Pagel CN Wasgewatte Wijesinghe DK Taghavi Esfandouni N Mackie EJ. Osteopontin, inflammation and myogenesis: influencing regeneration, fibrosis and size of skeletal muscle. J Cell Commun Signal (2014) 8(2):95–103. doi: 10.1007/s12079-013-0217-3 24318932 PMC4063988

[47] Vetrone SA Montecino-Rodriguez E Kudryashova E Kramerova I Hoffman EP Liu SD. Osteopontin promotes fibrosis in dystrophic mouse muscle by modulating immune cell subsets and intramuscular TGF-beta. J Clin Invest (2009) 119(6):1583–94. doi: 10.1172/jci37662 PMC2689112 19451692

[48] Paliwal P Pishesha N Wijaya D Conboy IM. Age dependent increase in the levels of osteopontin inhibits skeletal muscle regeneration. Aging (2012) 4(8):553–66. doi: 10.18632/aging.100477 PMC3461343 22915705

[49] Luukkonen J Hilli M Nakamura M Ritamo I Valmu L Kauppinen K. Osteoclasts secrete osteopontin into resorption lacunae during bone resorption. Histochem Cell Biol (2019) 151(6):475–87. doi: 10.1007/s00418-019-01770-y PMC6542781 30637455

[50] Singh A Gill G Kaur H Amhmed M Jakhu H. Role of osteopontin in bone remodeling and orthodontic tooth movement: A review. Prog Orthod (2018) 19(1):18. doi: 10.1186/s40510-018-0216-2 29938297 PMC6015792

[51] Carvalho MS Poundarik AA Cabral JMS da Silva CL Vashishth D. Biomimetic matrices for rapidly forming mineralized bone tissue based on stem cell-mediated osteogenesis. Sci Rep (2018) 8(1):14388. doi: 10.1038/s41598-018-32794-4 30258220 PMC6158243

[52] Chang IC Chiang TI Yeh KT Lee H Cheng YW. Increased serum osteopontin is a risk factor for osteoporosis in menopausal women. Osteoporos Int (2010) 21(8):1401–9. doi: 10.1007/s00198-009-1107-7 20238102

[53] Sroga GE Vashishth D. Phosphorylation of extracellular bone matrix proteins and its contribution to bone fragility. J Bone Miner Res (2018) 33(12):2214–29. doi: 10.1002/jbmr.3552 30001467

[54] Kavukcuoglu NB Denhardt DT Guzelsu N Mann AB. Osteopontin deficiency and aging on nanomechanics of mouse bone. J BioMed Mater Res A (2007) 83(1):136–44. doi: 10.1002/jbm.a.31081 17390367

[55] Matsui Y Iwasaki N Kon S Takahashi D Morimoto J Matsui Y. Accelerated development of aging-associated and instability-induced osteoarthritis in osteopontin-deficient mice. Arthritis Rheum (2009) 60(8):2362–71. doi: 10.1002/art.24705 19644889

[56] Ishijima M Rittling SR Yamashita T Tsuji K Kurosawa H Nifuji A. Enhancement of osteoclastic bone resorption and suppression of osteoblastic bone formation in response to reduced mechanical stress do not occur in the absence of osteopontin. J Exp Med (2001) 193(3):399–404. doi: 10.1084/jem.193.3.399 11157060 PMC2195919

[57] Ishijima M Ezura Y Tsuji K Rittling SR Kurosawa H Denhardt DT. Osteopontin is associated with nuclear factor kappaB gene expression during tail-suspension-induced bone loss. Exp Cell Res (2006) 312(16):3075–83. doi: 10.1016/j.yexcr.2006.06.003 16889770

[58] Rawji KS Mishra MK Michaels NJ Rivest S Stys PK Yong VW. Immunosenescence of microglia and macrophages: Impact on the ageing central nervous system. Brain (2016) 139(Pt 3):653–61. doi: 10.1093/brain/awv395 PMC5839598 26912633

[59] Madore C Yin Z Leibowitz J Butovsky O. Microglia, lifestyle stress, and neurodegeneration. Immunity (2020) 52(2):222–40. doi: 10.1016/j.immuni.2019.12.003 PMC7234821 31924476

[60] Yang T Sun Y Lu Z Leak RK Zhang F. The impact of cerebrovascular aging on vascular cognitive impairment and dementia. Ageing Res Rev (2017) 34:15–29. doi: 10.1016/j.arr.2016.09.007 27693240 PMC5250548

[61] Baidya F Bohra M Datta A Sarmah D Shah B Jagtap P. Neuroimmune crosstalk and evolving pharmacotherapies in neurodegenerative diseases. Immunology (2021) 162(2):160–78. doi: 10.1111/imm.13264 PMC7808166 32939758

[62] Voet S Srinivasan S Lamkanfi M van Loo G. Inflammasomes in neuroinflammatory and neurodegenerative diseases. EMBO Mol Med (2019) 11(6):e10248. doi: 10.15252/emmm.201810248 31015277 PMC6554670

[63] Singh A Kukreti R Saso L Kukreti S. Oxidative stress: A key modulator in neurodegenerative diseases. Molecules (2019) 24(8):1583. doi: 10.3390/molecules24081583 31013638 PMC6514564

[64] Hetz C Saxena S. ER stress and the unfolded protein response in neurodegeneration. Nat Rev Neurol (2017) 13(8):477–91. doi: 10.1038/nrneurol.2017.99 28731040

[65] Johnson J Mercado-Ayon E Mercado-Ayon Y Dong YN Halawani S Ngaba L. Mitochondrial dysfunction in the development and progression of neurodegenerative diseases. Arch Biochem Biophys (2021) 702:108698. doi: 10.1016/j.abb.2020.108698 33259796

[66] Luo F Sandhu AF Rungratanawanich W Williams GE Akbar M Zhou S. Melatonin and autophagy in aging-related neurodegenerative diseases. Int J Mol Sci (2020) 21(19):7174. doi: 10.3390/ijms21197174 32998479 PMC7584015

[67] Harry GJ. Microglia during development and aging. Pharmacol Ther (2013) 139(3):313–26. doi: 10.1016/j.pharmthera.2013.04.013 PMC3737416 23644076

[68] Wang JL Xu CJ. Astrocytes autophagy in aging and neurodegenerative disorders. BioMed Pharmacother (2020) 122:109691. doi: 10.1016/j.biopha.2019.109691 31786465

[69] Kapoor A Nation DA. Role of notch signaling in neurovascular aging and alzheimer's disease. Semin Cell Dev Biol (2021) 116:90–7. doi: 10.1016/j.semcdb.2020.12.011 PMC8236496 33384205

[70] Mangiola F Nicoletti A Gasbarrini A Ponziani FR. Gut microbiota and aging. Eur Rev Med Pharmacol Sci (2018) 22(21):7404–13. doi: 10.26355/eurrev_201811_16280 30468488

[71] Xu M Mo X Huang H Chen X Liu H Peng Z. Yeast β-glucan alleviates cognitive deficit by regulating gut microbiota and metabolites in Aβ(1)(-)(42)-induced AD-like mice. Int J Biol Macromol (2020) 161:258–70. doi: 10.1016/j.ijbiomac.2020.05.180 32522544

[72] Roos PM. Osteoporosis in neurodegeneration. J Trace Elem Med Biol (2014) 28(4):418–21. doi: 10.1016/j.jtemb.2014.08.010 25220531

[73] Gliem M Krammes K Liaw L van Rooijen N Hartung HP Jander S. Macrophage-derived osteopontin induces reactive astrocyte polarization and promotes re-establishment of the blood brain barrier after ischemic stroke. Glia (2015) 63(12):2198–207. doi: 10.1002/glia.22885 26148976

[74] Chan JL Reeves TM Phillips LL. Osteopontin expression in acute immune response mediates hippocampal synaptogenesis and adaptive outcome following cortical brain injury. Exp Neurol (2014) 261:757–71. doi: 10.1016/j.expneurol.2014.08.015 PMC4262258 25151457

[75] Sun Y Yin XS Guo H Han RK He RD Chi LJ. Elevated osteopontin levels in mild cognitive impairment and alzheimer's disease. Mediators Inflammation (2013) 2013:615745. doi: 10.1155/2013/615745 PMC3612435 23576854

[76] Lin Y Zhou M Dai W Guo W Qiu J Zhang Z. Bone-derived factors as potential biomarkers for parkinson's disease. Front Aging Neurosci (2021) 13:634213. doi: 10.3389/fnagi.2021.634213 33732138 PMC7959739

[77] Maetzler W Berg D Schalamberidze N Melms A Schott K Mueller JC. Osteopontin is elevated in parkinson's disease and its absence leads to reduced neurodegeneration in the MPTP model. Neurobiol Dis (2007) 25(3):473–82. doi: 10.1016/j.nbd.2006.10.020 17188882

[78] Carecchio M Comi C. The role of osteopontin in neurodegenerative diseases. J Alzheimers Dis (2011) 25(2):179–85. doi: 10.3233/JAD-2011-102151 21358042

[79] Iczkiewicz J Jackson MJ Smith LA Rose S Jenner P. Osteopontin expression in substantia nigra in MPTP-treated primates and in parkinson's disease. Brain Res (2006) 1118(1):239–50. doi: 10.1016/j.brainres.2006.08.036 16962083

[80] Broom L Jenner P Rose S. Increased neurotrophic factor levels in ventral mesencephalic cultures do not explain the protective effect of osteopontin and the synthetic 15-mer RGD domain against MPP+ toxicity. Exp Neurol (2015) 263:1–7. doi: 10.1016/j.expneurol.2014.09.005 25218309

[81] Iczkiewicz J Broom L Cooper JD Wong AM Rose S Jenner P. The RGD-containing peptide fragment of osteopontin protects tyrosine hydroxylase positive cells against toxic insult in primary ventral mesencephalic cultures and in the rat substantia nigra. J Neurochem (2010) 114(6):1792–804. doi: 10.1111/j.1471-4159.2010.06896.x 20626561

[82] Comi C Carecchio M Chiocchetti A Nicola S Galimberti D Fenoglio C. Osteopontin is increased in the cerebrospinal fluid of patients with alzheimer's disease and its levels correlate with cognitive decline. J Alzheimers Dis (2010) 19(4):1143–8. doi: 10.3233/jad-2010-1309 20308780

[83] Wung JK Perry G Kowalski A Harris PL Bishop GM Trivedi MA. Increased expression of the remodeling- and tumorigenic-associated factor osteopontin in pyramidal neurons of the alzheimer's disease brain. Curr Alzheimer Res (2007) 4(1):67–72. doi: 10.2174/156720507779939869 17316167

[84] Chai YL Chong JR Raquib AR Xu X Hilal S Venketasubramanian N. Plasma osteopontin as a biomarker of alzheimer's disease and vascular cognitive impairment. Sci Rep (2021) 11(1):4010. doi: 10.1038/s41598-021-83601-6 33597603 PMC7889621

[85] Wirths O Breyhan H Marcello A Cotel MC Bruck W Bayer TA. Inflammatory changes are tightly associated with neurodegeneration in the brain and spinal cord of the APP/PS1KI mouse model of alzheimer's disease. Neurobiol Aging (2010) 31(5):747–57. doi: 10.1016/j.neurobiolaging.2008.06.011 18657882

[86] Rentsendorj A Sheyn J Fuchs DT Daley D Salumbides BC Schubloom HE. A novel role for osteopontin in macrophage-mediated amyloid-β clearance in alzheimer's models. Brain Behav Immun (2018) 67:163–80. doi: 10.1016/j.bbi.2017.08.019 PMC5865478 28860067

[87] Zhao C Fancy SP ffrench-Constant C Franklin RJ. Osteopontin is extensively expressed by macrophages following CNS demyelination but has a redundant role in remyelination. Neurobiol Dis (2008) 31(2):209–17. doi: 10.1016/j.nbd.2008.04.007 18539470

[88] Hasegawa-Ishii S Takei S Inaba M Umegaki H Chiba Y Furukawa A. Defects in cytokine-mediated neuroprotective glial responses to excitotoxic hippocampal injury in senescence-accelerated mouse. Brain Behav Immun (2011) 25(1):83–100. doi: 10.1016/j.bbi.2010.08.006 20804842

[89] Lin YH Huang CJ Chao JR Chen ST Lee SF Young-Yen JJ. Coupling of osteopontin and its cell surface receptor CD44 to the cell survival response elicited by interleukin-3 or granulocyte-macrophage colony-stimulating factor. Mol Cell Biol (2000) 20(8):2734–42. doi: 10.1128/MCB.20.8.2734-2742.2000 PMC85489 10733576

[90] Izrael M Slutsky SG Admoni T Cohen L Granit A Hasson A. Safety and efficacy of human embryonic stem cell-derived astrocytes following intrathecal transplantation in SOD1(G93A) and NSG animal models. Stem Cell Res Ther (2018) 9(1):152. doi: 10.1186/s13287-018-0890-5 29871694 PMC5989413

[91] Rockey DC Bell PD Hill JA. Fibrosis–a common pathway to organ injury and failure. N Engl J Med (2015) 372(12):1138–49. doi: 10.1056/NEJMra1300575 25785971

[92] Shirakabe A Ikeda Y Sciarretta S Zablocki DK Sadoshima J. Aging and autophagy in the heart. Circ Res (2016) 118(10):1563–76. doi: 10.1161/CIRCRESAHA.116.307474 PMC4869999 27174950

[93] Kurose H. Cardiac fibrosis and fibroblasts. Cells (2021) 10(7):1716. doi: 10.3390/cells10071716 34359886 PMC8306806

[94] Singh M Foster CR Dalal S Singh K. Osteopontin: role in extracellular matrix deposition and myocardial remodeling post-MI. J Mol Cell Cardiol (2010) 48(3):538–43. doi: 10.1016/j.yjmcc.2009.06.015 PMC2823840 19573532

[95] Collins AR Schnee J Wang W Kim S Fishbein MC Bruemmer D. Osteopontin modulates angiotensin II-induced fibrosis in the intact murine heart. J Am Coll Cardiol (2004) 43(9):1698–705. doi: 10.1016/j.jacc.2003.11.058 15120833

[96] Matsui Y Jia N Okamoto H Kon S Onozuka H Akino M. Role of osteopontin in cardiac fibrosis and remodeling in angiotensin II-induced cardiac hypertrophy. Hypertension (2004) 43(6):1195–201. doi: 10.1161/01.HYP.0000128621.68160.dd 15123578

[97] Satoh M Nakamura M Akatsu T Shimoda Y Segawa I Hiramori K. Myocardial osteopontin expression is associated with collagen fibrillogenesis in human dilated cardiomyopathy. Eur J Heart Fail (2005) 7(5):755–62. doi: 10.1016/j.ejheart.2004.10.019 16087132

[98] Yu Q Vazquez R Khojeini EV Patel C Venkataramani R Larson DF. IL-18 induction of osteopontin mediates cardiac fibrosis and diastolic dysfunction in mice. Am J Physiol Heart Circ Physiol (2009) 297(1):H76–85. doi: 10.1152/ajpheart.01285.2008 PMC2711747 19429811

[99] Tanino A Okura T Nagao T Kukida M Pei Z Enomoto D. Interleukin-18 deficiency protects against renal interstitial fibrosis in aldosterone/salt-treated mice. Clin Sci (Lond) (2016) 130(19):1727–39. doi: 10.1042/cs20160183 27413021

[100] Lorenzen JM Schauerte C Hübner A Kölling M Martino F Scherf K. Osteopontin is indispensible for AP1-mediated angiotensin II-related miR-21 transcription during cardiac fibrosis. Eur Heart J (2015) 36(32):2184–96. doi: 10.1093/eurheartj/ehv109 PMC4543785 25898844

[101] Pollard CM Desimine VL Wertz SL Perez A Parker BM Maning J. Deletion of osteopontin enhances β_2_-adrenergic receptor-dependent anti-fibrotic signaling in cardiomyocytes. Int J Mol Sci (2019) 20(6):1396. doi: 10.3390/ijms20061396 30897705 PMC6470638

[102] Sun SN Ni SH Li Y Liu X Deng JP Chen ZX. G-MDSCs promote aging-related cardiac fibrosis by activating myofibroblasts and preventing senescence. Cell Death Dis (2021) 12(6):594. doi: 10.1038/s41419-021-03874-7 34103476 PMC8187421

[103] Sawaki D Czibik G Pini M Ternacle J Suffee N Mercedes R. Visceral adipose tissue drives cardiac aging through modulation of fibroblast senescence by osteopontin production. Circulation (2018) 138(8):809–22. doi: 10.1161/circulationaha.117.031358 29500246

[104] Shirakawa K Yan X Shinmura K Endo J Kataoka M Katsumata Y. Obesity accelerates T cell senescence in murine visceral adipose tissue. J Clin Invest (2016) 126(12):4626–39. doi: 10.1172/jci88606 PMC5127667 27820698

[105] Lin R Wu S Zhu D Qin M Liu X. Osteopontin induces atrial fibrosis by activating Akt/GSK-3β/β-catenin pathway and suppressing autophagy. Life Sci (2020) 245:117328. doi: 10.1016/j.lfs.2020.117328 31954162

[106] de Picciotto NE Gano LB Johnson LC Martens CR Sindler AL Mills KF. Nicotinamide mononucleotide supplementation reverses vascular dysfunction and oxidative stress with aging in mice. Aging Cell (2016) 15(3):522–30. doi: 10.1111/acel.12461 PMC4854911 26970090

[107] Ungvari Z Tarantini S Donato AJ Galvan V Csiszar A. Mechanisms of vascular aging. Circ Res (2018) 123(7):849–67. doi: 10.1161/circresaha.118.311378 PMC6248882 30355080

[108] Ungvari Z Tarantini S Sorond F Merkely B Csiszar A. Mechanisms of vascular aging, a geroscience perspective: JACC focus seminar. J Am Coll Cardiol (2020) 75(8):931–41. doi: 10.1016/j.jacc.2019.11.061 PMC8559983 32130929

[109] Mohler ER 3rd Adam LP McClelland P Graham L Hathaway DR. Detection of osteopontin in calcified human aortic valves. Arterioscler Thromb Vasc Biol (1997) 17(3):547–52. doi: 10.1161/01.atv.17.3.547 9102175

[110] Hirota S Imakita M Kohri K Ito A Morii E Adachi S. Expression of osteopontin messenger RNA by macrophages in atherosclerotic plaques. a possible association with calcification. Am J Pathol (1993) 143(4):1003–8. PMC1887054 8213995

[111] Ortega MA Asúnsolo Á Pekarek L Alvarez-Mon MA Delforge A Sáez MA. Histopathological study of JNK in venous wall of patients with chronic venous insufficiency related to osteogenesis process. Int J Med Sci (2021) 18(9):1921–34. doi: 10.7150/ijms.54052 PMC8040408 33850461

[112] Schinzari F Tesauro M Bertoli A Valentini A Veneziani A Campia U. Calcification biomarkers and vascular dysfunction in obesity and type 2 diabetes: influence of oral hypoglycemic agents. Am J Physiol Endocrinol Metab (2019) 317(4):E658–e66. doi: 10.1152/ajpendo.00204.2019 31408377

[113] Aryan M Kepez A Atalar E Hazirolan T Haznedaroglu I Akata D. Association of plasma osteopontin levels with coronary calcification evaluated by tomographic coronary calcium scoring. J Bone Miner Metab (2009) 27(5):591–7. doi: 10.1007/s00774-009-0078-2 19365701

[114] Speer MY Chien YC Quan M Yang HY Vali H McKee MD. Smooth muscle cells deficient in osteopontin have enhanced susceptibility to calcification. vitro Cardiovasc Res (2005) 66(2):324–33. doi: 10.1016/j.cardiores.2005.01.023 15820201

[115] Cai Y Teng X Pan CS Duan XH Tang CS Qi YF. Adrenomedullin up-regulates osteopontin and attenuates vascular calcification *via* the cAMP/PKA signaling pathway. Acta Pharmacol Sin (2010) 31(10):1359–66. doi: 10.1038/aps.2010.89 PMC4012911 20802507

[116] Ohri R Tung E Rajachar R Giachelli CM. Mitigation of ectopic calcification in osteopontin-deficient mice by exogenous osteopontin. Calcif Tissue Int (2005) 76(4):307–15. doi: 10.1007/s00223-004-0071-7 15812576

[117] Speer MY McKee MD Guldberg RE Liaw L Yang HY Tung E. Inactivation of the osteopontin gene enhances vascular calcification of matrix gla protein-deficient mice: Evidence for osteopontin as an inducible inhibitor of vascular calcification. vivo J Exp Med (2002) 196(8):1047–55. doi: 10.1084/jem.20020911 PMC2194039 12391016

[118] Zhang L Xu Z Wu Y Liao J Zeng F Shi L. Akt/eNOS and MAPK signaling pathways mediated the phenotypic switching of thoracic aorta vascular smooth muscle cells in aging/hypertensive rats. Physiol Res (2018) 67(4):543–53. doi: 10.33549/physiolres.933779 29750880

[119] Shao JS Sierra OL Cohen R Mecham RP Kovacs A Wang J. Vascular calcification and aortic fibrosis: A bifunctional role for osteopontin in diabetic arteriosclerosis. Arterioscler Thromb Vasc Biol (2011) 31(8):1821–33. doi: 10.1161/ATVBAHA.111.230011 PMC3141097 21597007

[120] Ding M Zhang Q Zhang M Jiang X Wang M Ni L. Phosphate overload stimulates inflammatory reaction *via* PiT-1 and induces vascular calcification in uremia. J Ren Nutr (2021) 32(2):178–88. doi: 10.1053/j.jrn.2021.03.008 34688540

[121] Gericke A Qin C Spevak L Fujimoto Y BuTLER WT Sørensen ES. Importance of phosphorylation for osteopontin regulation of biomineralization. Calcif Tissue Int (2005) 77(1):45–54. doi: 10.1007/s00223-004-1288-1 16007483 PMC1451414

[122] Grau JB Poggio P Sainger R Vernick WJ Seefried WF Branchetti E. Analysis of osteopontin levels for the identification of asymptomatic patients with calcific aortic valve disease. Ann Thorac Surg (2012) 93(1):79–86. doi: 10.1016/j.athoracsur.2011.08.036 22093695 PMC3269243

[123] Jono S Peinado C Giachelli CM. Phosphorylation of osteopontin is required for inhibition of vascular smooth muscle cell calcification. J Biol Chem (2000) 275(26):20197–203. doi: 10.1074/jbc.M909174199 10766759

[124] Li J Carrillo García C Riedt T Brandes M Szczepanski S Brossart P. Murine hematopoietic stem cell reconstitution potential is maintained by osteopontin during aging. Sci Rep (2018) 8(1):2833. doi: 10.1038/s41598-018-21324-x 29434282 PMC5809550

[125] Guidi N Sacma M Ständker L Soller K Marka G Eiwen K. Osteopontin attenuates aging-associated phenotypes of hematopoietic stem cells. EMBO J (2017) 36(7):840–53. doi: 10.15252/embj.201694969 PMC5376966 28254837

[126] Magdaleno F Ge X Fey H Lu Y Gaskell H Blajszczak CC. Osteopontin deletion drives hematopoietic stem cell mobilization to the liver and increases hepatic iron contributing to alcoholic liver disease. Hepatol Commun (2018) 2(1):84–98. doi: 10.1002/hep4.1116 29404515 PMC5776866

[127] Huda N Liu G Hong H Yan S Khambu B Yin XM. Hepatic senescence, the good and the bad. World J Gastroenterol (2019) 25(34):5069–81. doi: 10.3748/wjg.v25.i34.5069 PMC6747293 31558857

[128] Zhong W Wang X Rao Z Pan X Sun Y Jiang T. Aging aggravated liver ischemia and reperfusion injury by promoting hepatocyte necroptosis in an endoplasmic reticulum stress-dependent manner. Ann Transl Med (2020) 8(14):869. doi: 10.21037/atm-20-2822 32793713 PMC7396804

[129] Bopp A Wartlick F Henninger C Kaina B Fritz G. Rac1 modulates acute and subacute genotoxin-induced hepatic stress responses, fibrosis and liver aging. Cell Death Dis (2013) 4(3):e558. doi: 10.1038/cddis.2013.57 23519127 PMC3613835

[130] Kane AE Mitchell SJ Mach J Huizer-Pajkos A McKenzie C Jones B. Acetaminophen hepatotoxicity in mice: Effect of age, frailty and exposure type. Exp Gerontol (2016) 73:95–106. doi: 10.1016/j.exger.2015.11.013 26615879 PMC5976491

[131] Michalopoulos GK Bhushan B. Liver regeneration: biological and pathological mechanisms and implications. Nat Rev Gastroenterol Hepatol (2021) 18(1):40–55. doi: 10.1038/s41575-020-0342-4 32764740

[132] Yagi S Hirata M Miyachi Y Uemoto S. Liver regeneration after hepatectomy and partial liver transplantation. Int J Mol Sci (2020) 21(21):8414. doi: 10.3390/ijms21218414 33182515 PMC7665117

[133] Urtasun R Lopategi A George J Leung TM Lu Y Wang X. Osteopontin, an oxidant stress sensitive cytokine, up-regulates collagen-I *via* integrin alpha(V)beta(3) engagement and PI3K/pAkt/NFkappaB signaling. Hepatology (2012) 55(2):594–608. doi: 10.1002/hep.24701 21953216 PMC3561739

[134] Leung TM Wang X Kitamura N Fiel MI Nieto N. Osteopontin delays resolution of liver fibrosis. Lab Invest (2013) 93(10):1082–9. doi: 10.1038/labinvest.2013.104 23999249

[135] Wang G Li X Chen S Zhao W Yang J Chang C. Expression profiles uncover the correlation of OPN signaling pathways with rat liver regeneration at cellular level. Cell Biol Int (2015) 39(11):1329–40. doi: 10.1002/cbin.10523 26269331

[136] Xu F Hua C Tautenhahn HM Dirsch O Dahmen U. The role of autophagy for the regeneration of the aging liver. Int J Mol Sci (2020) 21(10):3606. doi: 10.3390/ijms21103606 32443776 PMC7279469

[137] Weiskirchen R Tacke F. Relevance of autophagy in parenchymal and non-parenchymal liver cells for health and disease. Cells (2019) 8(1):16. doi: 10.3390/cells8010016 30609663 PMC6357193

[138] Liu A Guo E Yang J Yang Y Liu S Jiang X. Young plasma reverses age-dependent alterations in hepatic function through the restoration of autophagy. Aging Cell (2018) 17(1):e12708. doi: 10.1111/acel.12708 29210183 PMC5770779

[139] Escobar KA Cole NH Mermier CM VanDusseldorp TA. Autophagy and aging: Maintaining the proteome through exercise and caloric restriction. Aging Cell (2019) 18(1):e12876. doi: 10.1111/acel.12876 30430746 PMC6351830

[140] Chun Y Kim J. Autophagy: An essential degradation program for cellular homeostasis and life. Cells (2018) 7(12):278. doi: 10.3390/cells7120278 30572663 PMC6315530

[141] Jia CJ Sun H Dai CL. Autophagy contributes to liver regeneration after portal vein ligation in rats. Med Sci Monit (2019) 25:5674–82. doi: 10.12659/msm.915404 PMC6686715 31364611

[142] Cabiati M Salvadori C Sapio A Burchielli S Carlucci L Moscato S. Aging and biomarkers: Transcriptional levels evaluation of Osteopontin/miRNA-181a axis in hepatic tissue of rats in different age ranges. Exp Gerontol (2020) 133:110879. doi: 10.1016/j.exger.2020.110879 32061643

[143] Birke MT Neumann C Birke K Kremers J Scholz M. Changes of osteopontin in the aqueous humor of the DBA2/J glaucoma model correlated with optic nerve and RGC degenerations. Invest Ophthalmol Vis Sci (2010) 51(11):5759–67. doi: 10.1167/iovs.10-5558 20574028

[144] Ruzafa N Pereiro X Aspichueta P Araiz J Vecino E. The retina of osteopontin deficient mice in aging. Mol Neurobiol (2018) 55(1):213–21. doi: 10.1007/s12035-017-0734-9 PMC5808060 28866734

[145] Lekwuwa M Choudhary M Lad EM Malek G. Osteopontin accumulates in basal deposits of human eyes with age-related macular degeneration and may serve as a biomarker of aging. Mod Pathol (2021) 35(2):165–76. doi: 10.1038/s41379-021-00887-7 PMC8786662 34389792

[146] Linton PJ Dorshkind K. Age-related changes in lymphocyte development and function. Nat Immunol (2004) 5(2):133–9. doi: 10.1038/ni1033 14749784

[147] Finkel T Serrano M Blasco MA. The common biology of cancer and ageing. Nature (2007) 448(7155):767–74. doi: 10.1038/nature05985 17700693

[148] Haynes L Lefebvre JS. Age-related deficiencies in antigen-specific CD4 T cell responses: Lessons from mouse models. Aging Dis (2011) 2(5):374–81. PMC3295078 22396889

[149] Shimatani K Nakashima Y Hattori M Hamazaki Y Minato N. PD-1+ memory phenotype CD4+ T cells expressing C/EBPalpha underlie T cell immunodepression in senescence and leukemia. Proc Natl Acad Sci U.S.A. (2009) 106(37):15807–12. doi: 10.1073/pnas.0908805106 PMC2739871 19805226

[150] Xiong Y Wang Y Zhang J Zhao N Zhang H Zhang A. hPMSCs protects against d-galactose-induced oxidative damage of CD4(+) T cells through activating akt-mediated Nrf2 antioxidant signaling. Stem Cell Res Ther (2020) 11(1):468. doi: 10.1186/s13287-020-01993-0 33148324 PMC7641865

[151] Xiong Y Xiong Y Zhang H Zhao Y Han K Zhang J. hPMSCs-derived exosomal miRNA-21 protects against aging-related oxidative damage of CD4(+) T cells by targeting the PTEN/PI3K-Nrf2 axis. Front Immunol (2021) 12:780897. doi: 10.3389/fimmu.2021.780897 34887868 PMC8649962

